# Identification of the key flavonoid and lipid synthesis proteins in the pulp of two sea buckthorn cultivars at different developmental stages

**DOI:** 10.1186/s12870-022-03688-5

**Published:** 2022-06-17

**Authors:** Wei Du, Jian Ding, Shunguang Lu, Xiufeng Wen, Jianzhong Hu, Chengjiang Ruan

**Affiliations:** 1 Institute of Plant Resources, Key Laboratory of Biotechnology and Bioresources Utilization, Ministry of Education, Dalian Minzu University, Dalian, China; 2 Management Center of Seabuckthorn Development, Ministry of Water Resources, Beijing, China

**Keywords:** Sea buckthorn, TAG biosynthesis, Flavonoid biosynthesis, Cultivar variation, Proteomics

## Abstract

**Background:**

Sea buckthorn is an economically important woody plant for desertification control and water soil conservation. Its berry pulp is rich in flavonoids and unsaturated fatty acids. Cultivars containing high oil and flavonoid contents have higher economic value and will increase in the planting area. However, the cause of the differences in oil and flavonoid contents among cultivars is still unclear. The influence of key enzymes in the lipid and flavonoid synthesis pathways on their content needs to be explored and clarified.

**Results:**

The flavonoid content in XE (Xin’e 3) was 54% higher than that in SJ (Suiji 1). Rutin was the main flavonoid in sea buckthorn pulp, and the differences in the rutin content could cause flavonoid differences between the two cultivars. The oil content of XE was 31.58% higher than that of SJ, and the difference in oil content was highest at 50–70 DAF. High-throughput proteomics was used to quantify key enzymes of flavonoid and lipid synthesis pathways in two cultivars at three developmental stages. By functional annotation and KEGG analysis, 41 key enzymes related to phenylpropanoid biosynthesis, flavonoid biosynthesis, flavone and flavonol biosynthesis, fatty acid biosynthesis and TAG biosynthesis were quantified. CHS, F3H, ANS, fabD, FATA, FAB2, LPIN and plcC showed significant differences between the two cultivars. In addition, we quantified 6 oleosins. With the exception of a 16 kDa oleosin, the other oleosins in the two cultivars were positively correlated with oil content.

**Conclusions:**

In the flavonoid synthesis pathway, CHS and F3H were the main enzymes responsible for the difference in flavonoid content between the two cultivars. In the lipid synthesis pathway, LPIN, plcC and MGD were the main enzymes with different contents in the middle to late stages. Higher contents of LPIN and plcC in XE than in SJ could cause DAG to generate TAG from PC, since the difference in DGAT between the two cultivars was not significant. Investigating the causes of flavonoid and oil content differences among different cultivars from the perspective of proteomics, could provide a basis for understanding the regulatory mechanism of flavonoids and lipid synthesis in sea buckthorn pulp.

**Supplementary Information:**

The online version contains supplementary material available at 10.1186/s12870-022-03688-5.

## Background

Sea buckthorn (*Hippophae* *rhamnoides* L.), which belongs to the genus *Hippophae* L. in the family Elaeagnaceae, is a deciduous shrub or small tree used for soil and water conservation that is widely distributed in the subtropical and cold temperate zones of Eurasia [1]. Ripe sea buckthorn berries are oval in shape and are usually yellow, orange or red in color. Sea buckthorn fruit has been called a “superfruit” because it contains more than 100 kinds of bioactive compounds, such as essential amino acids, vitamins, tocopherols, carotenoids, polyphenols, flavonoids, beneficial fatty acids and other active substances [2]. The content of total flavonoids in sea buckthorn pulp is up to 360 mg/kg, which is 2.5 times that of blueberry, 3.6 times that of apple and 4.5 times that of strawberry [3, 4]. In recent years, the chemical constituents, quality control and pharmacokinetics of sea buckthorn flavonoids have been studied. It has been confirmed that sea buckthorn flavonoids have wide therapeutic potential in treating metabolic diseases and clinical application [5]. The flavonoids found in sea buckthorn pulp can reduce the incidence of many chronic diseases, such as functional decline caused by oxidative damage and cardiovascular disease [6, 7]. In addition, unsaturated fatty acids in sea buckthorn oil have significant effects on improving cell activity and lowering blood lipids [8]. At present, there are many studies on flavonoids in the leaves of sea buckthorn [9–11], but as an edible part, the types and contents of flavonoids in the pulp of sea buckthorn are also worth studying [3]. Genetic improvement of flavonoid and oil production and accumulation in sea buckthorn berries has always been one of the objectives of sea buckthorn breeding.

Although the synthesis pathways of flavonoids and TAG are clearly understood, the regulatory mechanisms of some key proteins, such as CHS, ACCase, DGAT and PDAT have been gradually clarified [12, 13]. However, the key enzymatic differences in the expression and regulation of flavonoid and TAG metabolism among different cultivars of sea buckthorn are still not well understood. The contents of oils and flavonoids in dried sea buckthorn pulp were shown to vary greatly among different cultivars [14–16]. Many researchers have studied the synthesis process of flavonoids and oils in sea buckthorn. Tahira found that the expression levels of *CHS*, *F3H*, *DFR* and *LDOX* in the flavonoid synthesis pathway were upregulated in high-flavonoid 'RC-4' cultivars compared to low-flavonoid cultivars [17]. Comparative transcriptomic analysis of TAG synthesis in seeded and nonseeded tissues of *H.* *rhamnoides* revealed that higher expression of the source gene *GPD1* and sink genes *DGAT1* and *DGAT2* contributed to G3P eventually generating more TAG in berry pulp [18]. Despite the above realization, there is little information on the temporal and intervarietal accumulation of flavonoids and TAG during berry development. Furthermore, there are still no reports on flavonoid and lipid biosynthesis and regulatory enzymes in sea buckthorn at the protein level.

Enzymes are directly involved in plant cell composition and metabolism and have become an important target in the study of plant anabolic pathways [19]. Proteomics, as a way to study metabolic pathways, is an effective means to study the protein composition of cells, tissues or organisms and their changes [20]. At present, protein quantification technology based on mass spectrometry has been fully applied in the study of the plant proteome. Compared with the two-dimensional electrophoresis method popular in the early twenty-first century, tandem mass tag-mass spectrometry has the advantages of good repeatability, high throughput, and high sensitivity for low abundance proteins [21]. In a previous study, we focused on the analysis of changes in lipid synthesis pathway proteins in the XE cultivar at three developmental stages. The abundance of proteins related to fatty acid and TAG synthesis peaked and remained stable beyond 50 DAF (days after flowering) [22]. It takes approximately 100–120 days for sea buckthorn berries to grow and mature, and the contents of bioactive components remain stable at the late stage of development. The early and middle stages of sea buckthorn berry development (30, 50 and 70 DAF) are the critical periods for berry firmness and color transitions. In this study, two sea buckthorn cultivars, XE with high oil and flavonoid contents in the pulp and SJ with lower oil and flavonoid contents, were selected as materials to i) determine the differences in the contents of key enzymes involved in the flavonoid and lipid biosynthesis pathways at three different developmental stages and ii) reveal the reasons for the differences in flavonoid and oil contents between the two sea buckthorn cultivars from the perspective of proteomics. These data will improve our understanding of flavonoid and lipid metabolism in sea buckthorn berries and provide a scientific basis for the breeding of sea buckthorn cultivars with high oil and flavonoid contents.

## Results

### Pulp oil contents in developing berries

**Fig. 1:**
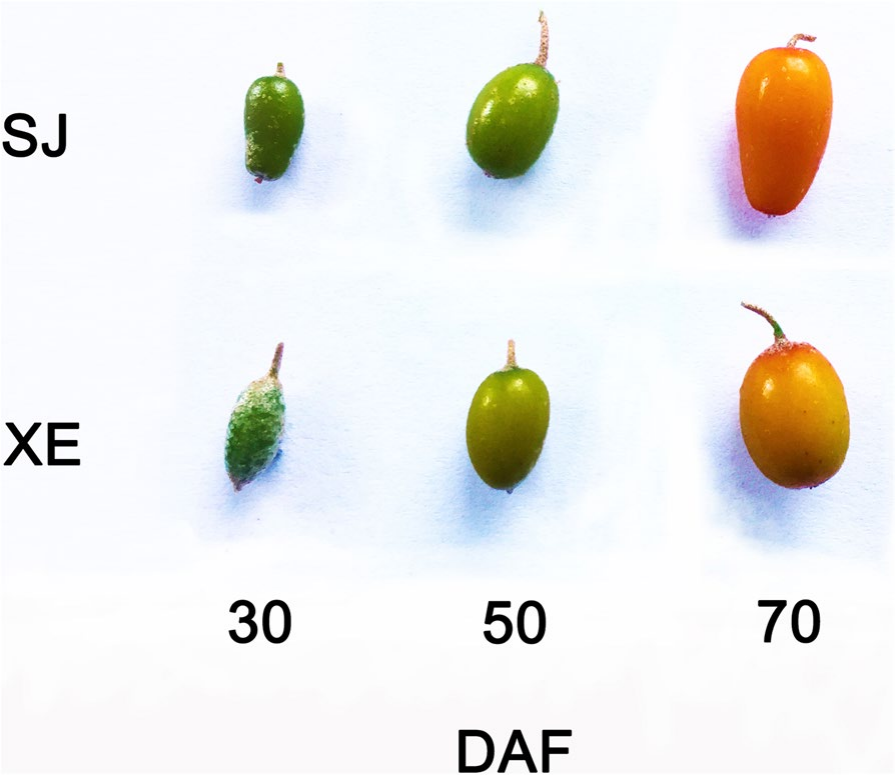
Changes in berry morphology during sea buckthorn development (berries at 30, 50, and 70 days after flowering (DAF)

**Fig. 2:**
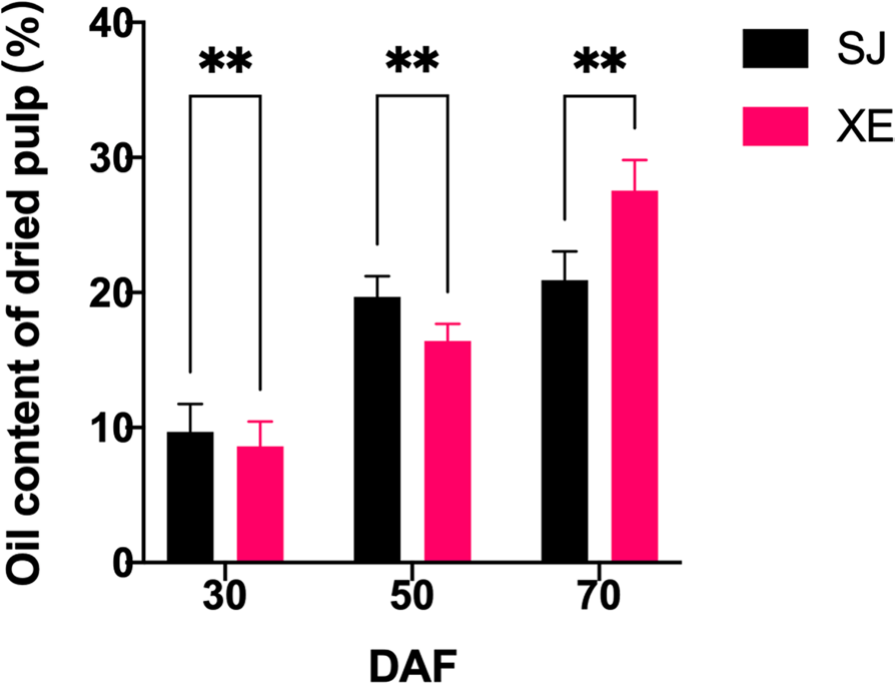
Oil contents in the pulp of sea buckthorn berries. * *p* < 0.05, ** *p* < 0.01, *** *p* < 0.001, **** *p* < 0.0001. All values represent the means of biological triplicates

Sea buckthorn berries were in the early stage of development until 30 DAF. The berry color was green, and berries were covered with white scaly hairs. In the early growth stage, a certain amount of oil accumulated in the pulp, but there was no significant difference in the pulp oil content between the two cultivars (Fig. 2). From 30 to 50 DAF, sea buckthorn berries entered the rapid growth period, where the berries became soft, their colour gradually changed from green to chartreuse, and the synthesis of pulp oil gradually accelerated (Fig. 1, Fig. 2). The pulp oil content of SJ was significantly higher than that of XE, which was 19.68% and 16.40%, respectively (*p* < 0.01). From 50 to 70 DAF, the scales of the outer skin of the berry disappeared, and the berry changed from chartreuse to orange. In terms of pulp oil content, the two cultivars showed large differences. The pulp oil content of the SJ only increased by approximately 1.3% between 50 and 70 DAF, while that of the XE maintained rapid growth and reached approximately 27.2%, which is approximately 1.4 times that of SJ, with significant differences (*p* < 0.0001). The results showed that the pulp oil content was significantly different between the two cultivars from 50 to 70 DAF.

### Flavonoid components in sea buckthorn pulp

The flavonoid components of sea buckthorn pulp at 70 DAF are shown in Table 1. The primary flavonoids in the pulp were rutin (C_27_H_30_O_16_), quercetin (C_15_H_10_O_7_), epigallocatechin (C_15_H_10_O_7_), isorhamnetin (C_16_H_12_O_7_), luteolin (C_15_H_10_O_6_) and naringin (C_27_H_32_O_14_). Kaempferol (C_15_H_10_O_6_) and gallocatechin gallate (C_22_H_18_O_11_) were not detected in the pulp. The flavonoid composition in the pulp of the two sea buckthorn cultivars was basically the same, but the contents varied. The flavonoid content in XE was 1.54 times higher than that in SJ, and the total flavonoid concentration was 38.98 μg/g. The difference in total flavonoids between the two cultivars was related to the rutin and quercetin contents, which had relatively high contents in the pulp. The rutin and quercetin contents in XE were 25.91 μg/g and 4.05 μg/g, respectively.

**Table 1:** Flavonoid contents in the pulp of sea buckthorn berries

**Flavonoid name**	SJ		XE	
	Content (μg/g)	Standard Error ±	Content (μg/g)	Standard Error ±
Isorhamnetin	0.460	0.031	4.051^**^	0.341
Quercetin	7.057	0.912	6.388	0.827
Epigallocatechin	1.952	0.314	2.262	0.439
Rutin	15.367	1.342	25.916^**^	1.544
Luteolin	0.357	0.062	0.360	0.021
Naringin	0.005	0	0.026^**^	0.001
Total	25.204	2.378	38.986^**^	3.261

* *p* < 0.05, ** *p* < 0.01. All values represent the means of three biological triplicates

### Proteomes at three developmental stages

**Fig. 3:**
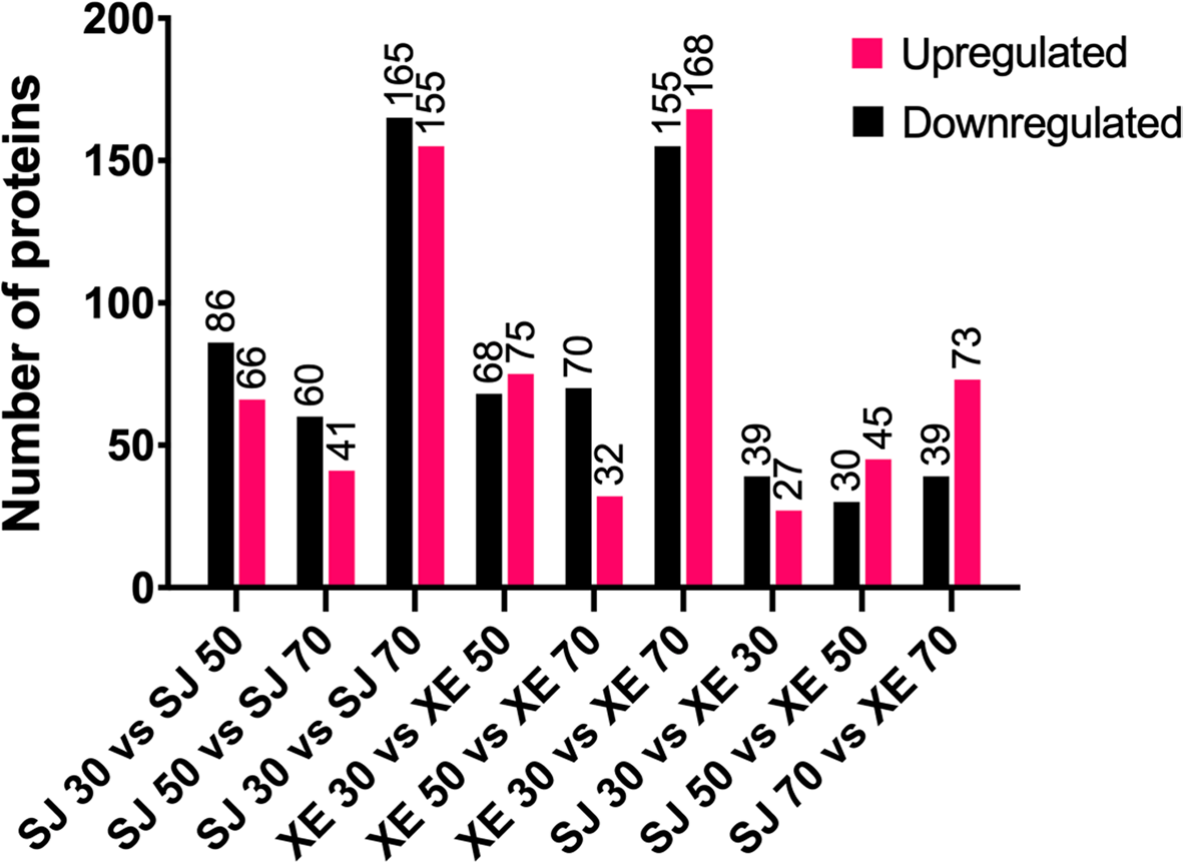
Number of differentially expressed proteins between SJ and XE cultiavrs at different berry developmental stages

In this study, more than 8600 proteins (unique peptides matched) were identified, among which 6170 were quantified simultaneously across all the samples and replicates. QC validation of MS data was shown in Fig. S2. Two comparisons were performed: proteins from the same cultivar at different developmental stages and those from different cultivars at the same stage. The number of proteins that were up and down-regulated was shown in Fig. 3. A total of 9 comparisons were made: differentially accumulated proteins with few changes (SJ *vs*. XE at 30, 50 and 70 DAF), many changes (30 *vs*. 70 DAF, XE and SJ), and an intermediate number of changes (30 *vs*. 50 DAF, 50 *vs*. 70 DAF, XE and SJ).

### Cluster analysis of differentially accumulated proteins

**Fig. 4:**
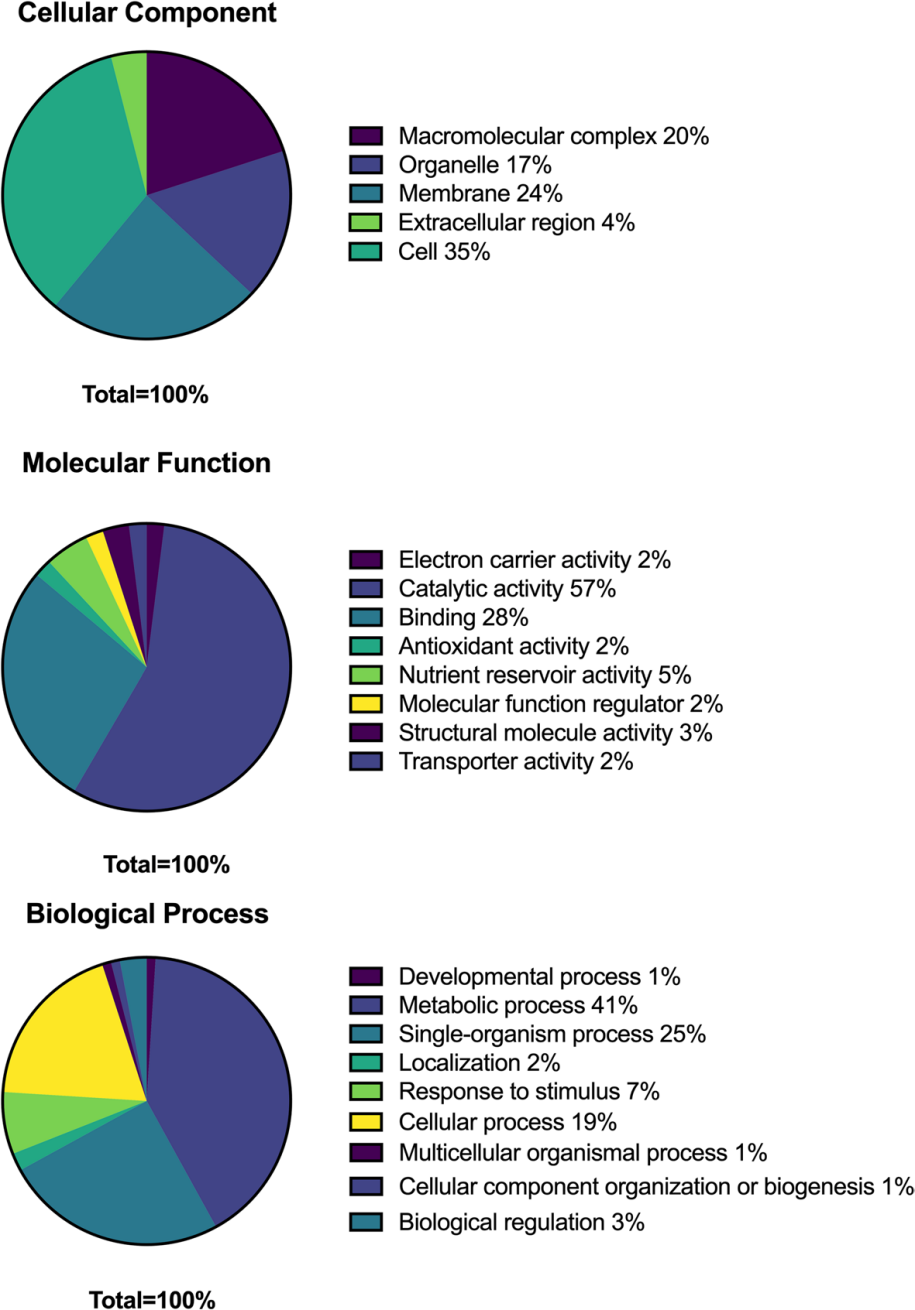
Differently expressed protein annotations for sea buckthorn berry (cellular components, molecular functions, and biological processes)

The significantly differentially accumulated proteins were classified into three categories using GO terms: biological process, cellular component and molecular function. The differentially accumulated proteins in the cellular component category were predominantly related to cells (35%), while 57% of the differentially accumulated proteins in molecular function were mainly involved in catalytic activity, and 41% of the differentially accumulated proteins in biological process were associated with metabolic processes. Figure 4 shows the annotations of above 1% differentially proteins for biological process, cellular component and molecular function.

**Fig. 5:**
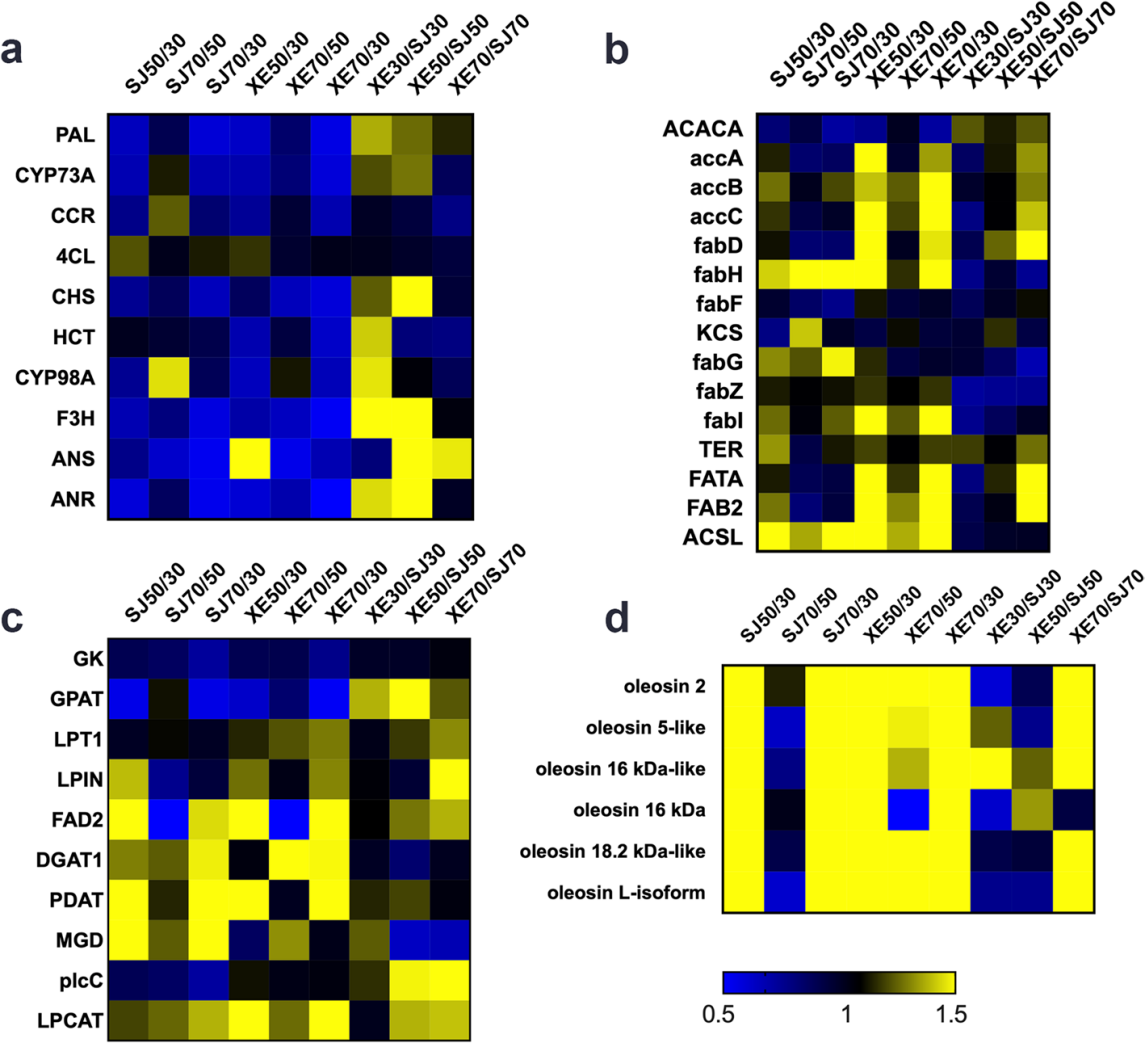
Changes in quantified proteins associated with the flavonoid synthesis pathway (A), fatty acid synthesis pathway (B), triacylglycerol synthesis pathway (C) and oleosin synthesis (D) between SJ and XE cultivars at different stages of sea buckthorn berry development

To determine the reasons for the high oil and high flavonoids contents in the pulp of the XE cultivar, the proteins related to flavonoids, fatty acids, TAG synthesis and oleosins were further enriched and analysed. Forty-one key enzymes were successfully annotated and quantified (Fig. 5; Table S4). The expression levels of proteins involved in flavonoid biosynthesis decreased with sea buckthorn berry development, and 50% of the proteins had significant expression differences between two sea buckthorn cultivars (Fig. 5a). Although fatty acid synthesis proteins varied greatly across different stages, only a small number of proteins showed differences at 70 DAF between two sea buckthorn cultivars (Fig. 5b). Most of the proteins involved in TAG synthesis were varied between different stages and cultivars (Fig. 5c). The expression levels of oleosins increased from 30 to 50 DAF in the two cultivars but showed significant differences between the two sea buckthorn cultivars at the late stage of berry development (Fig. 5d).

### Proteins involved in the flavonoid biosynthesis pathway

**Fig. 6:**
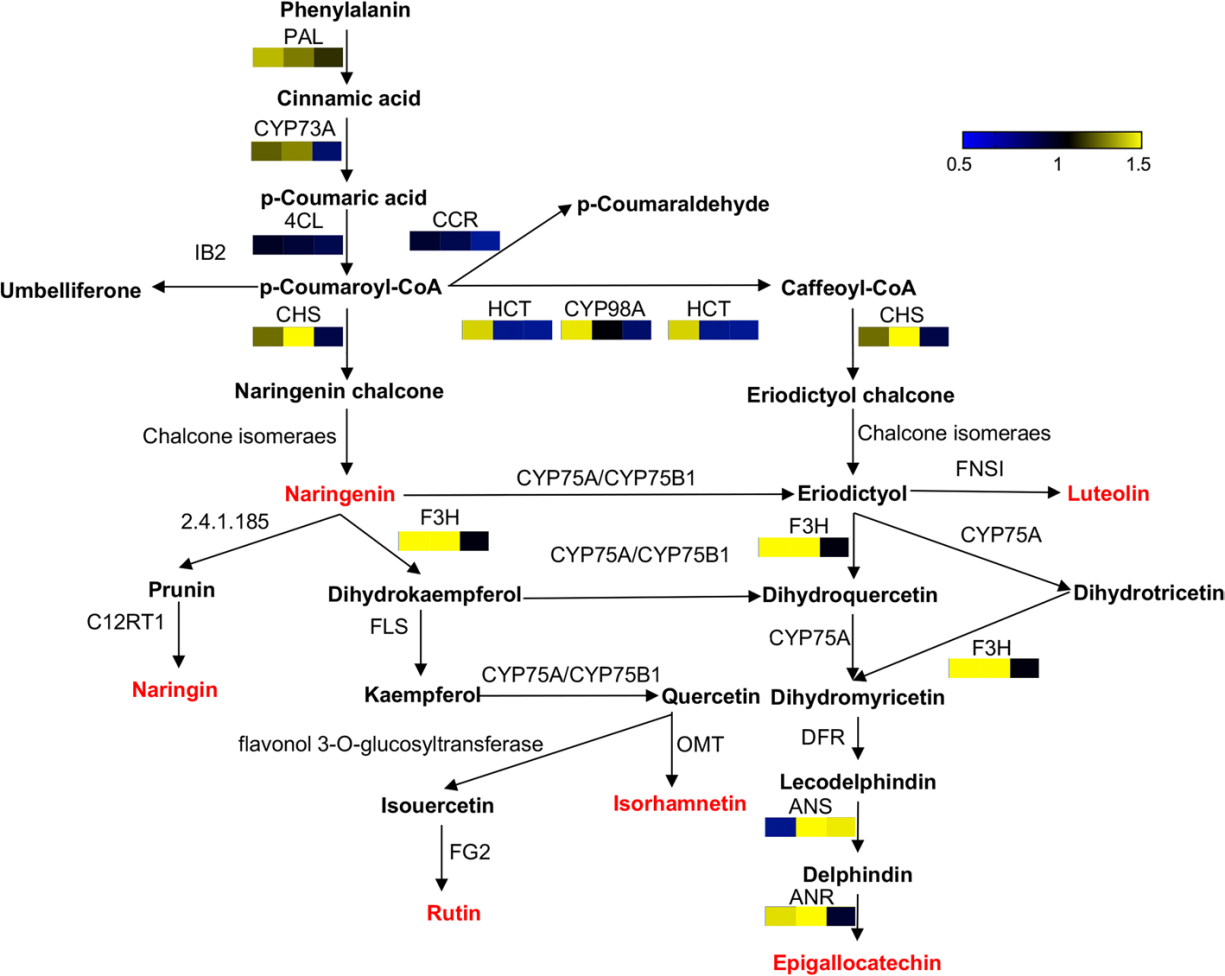
Comparison of key proteins in the flavonoid synthesis pathway between SJ and XE at different developmental stages. The pattern under each enzyme is representative of the ratio of the protein content between SJ and XE cultivars. Left: XE 30 DAF/SJ 30 DAF, middle XE 50 DAF/SJ 50 DAF, right: XE 70 DAF/SJ 70 DAF. This graph was modified from a KEGG map (ko00940, ko00941 and ko00944) [23]

In plant tissues, flavonoids are mainly synthesized from phenylalanine, with major compounds being aurones, flavanones, flavonols and isoflavonoids. Based on KEGG analysis, phenylpropanoid biosynthesis (map 00,940), flavonoid biosynthesis (map 00,941) and flavone and flavonol biosynthesis (map 00,944) were identified as participating in the flavonoid synthesis pathways in the pulp of sea buckthorn berries (Fig. 6) [23]. In contrast to the oil synthesis pathway, most of the proteins in the flavonoid synthesis pathway were higher in the early stage of berry development and then decreased gradually (Fig. 5A). The content of most proteins in XE was higher than that in the SJ at 30 DAF and 50 DAF and included those regulating PAL, CHS, CYP98A, F3H and ANR. Among them, CHS and F3H showed significant differences. ANS was upregulated from 30 to 50 DAF in XE, while 4CL exhibited stable abundance throughout berry development in XE and SJ.

### Proteins related to the fatty acid biosynthesis pathway

Based on functional annotation and the KEGG database, 15 kinds of proteins related to fatty acid biosynthesis were identified (Fig. 5B). At 30 DAF, one-third of the proteins were expressed at lower levels in XE than in SJ, including ACCase, beta-ketoacyl-acyl carrier protein synthase III (fabH), 3-hydroxyacyl-[acyl-carrier-protein] dehydratase (fabZ), enoyl-[acyl-carrier protein] reductase I (fabI) and FATA. Only fabZ showed significantly lower expression in XE than in SJ at 50 DAF, and the expression of the other proteins was not significantly different. At 70 DAF, acyl-[acyl-carrier-protein] desaturase (fadD), FAB2 and FATA in the fatty acid biosynthesis pathway were significantly upregulated in XE compared to SJ. The contents of fabH, 3-oxoacyl-[acyl-carrier protein] reductase (fabG) and fabZ were always slightly lower in XE than in SJ.

### Proteins involved in the TAG biosynthesis pathway

As the final step of lipid synthesis, the TAG synthesis pathway is different from the fatty acid polycyclic reaction. Protein changes in the two cultivars at three stages are shown in Fig. 5C. Glycerol kinase, PDAT and DGAT1 in XE and SJ remained at the same level from 30–70 DAF. MGD was downregulated in SJ at 50 and 70 DAF compared to its level in XE. Other proteins showed no significant difference between the two cultivars at 30 DAF but were significantly upregulated in XE with berry development from 50 to 70 DAF.

### Proteins related to oleosins

In this study, we successfully quantified six oleosins with molecular weights ranging from 15–20 kDa (Fig. 5D). The change in oleosin abundance was highly related to the change in the oil content, and the two cultivars showed different trends. Oleosin in SJ showed a significant increase from 30 to 50 DAF, and the average increase was approximately 4.61-fold. However, after 50 DAF, the amount of oleosin decreased rather than increased, and after 50 DAF, the amount of oil body protein did not increase, while and the content of oleosin 5-like and oleosin L-isoform decreased slightly. Compared with those in SJ, oleosins in XE still increased significantly during the period of 50–70 DAF. This result also showed that the difference in oleosin content between XE and SJ occurred during the period of 50–70 DAF.

### qPCR validation

To verify the mass spectrum results at the mRNA level, qPCR was performed on key enzymes of the flavonoid and lipid biosynthesis pathways that showed significant differences between XE and SJ at three developmental stages. Nine proteins of interest, namely, CHS, F3H, fabD, FATA, FAB2, GPAT, LPIN, MGD and plcC, were selected for further analysis (Table 2). The qPCR data indicated that *CHS*, *F3H* and *GPAT* exhibited common trends between XE and SJ based on mass spectrum data. The relatively high mRNA contents of *LPIN* and *plcC* at 50 DAF were reflected in the protein level at later stages in XE. Due to translation efficiency and posttranslation regulation, although the mRNA results were not completely consistent with the proteomics results, most of the proteins showed similar trends, which supported the plausibility and reliability of the mass spectrum data.

**Table 2:** Profiles of gene (qRT-PCR) and protein levels (TMT data) involved in flavonoid and lipid synthesis in sea buckthorn pulp at different developmental stages

Protein name	Definition	Protein level (TMT data)			Gene level (qRT-PCR)		
		**XE30/SJ30**	**XE50/SJ50**	**XE70/SJ70**	**XE30/SJ30**	**XE50/SJ50**	**XE70/SJ70**
CHS	chalcone synthase [EC:2.3.1.74]	1.87^a^	1.29	0.99	17.32^a^	4.99^a^	5.13^a^
F3H	flavanone 3-hydroxylase [EC:1.14.11.9]	1.55^a^	1.71^a^	0.95	14.82^a^	11.39^a^	0.77
fabD	[acyl-carrier-protein] S-malonyltransferase [EC:2.3.1.39]	0.78	1.20	1.58^a^	0.82	2.47^a^	1.11
FATA	fatty acyl-ACP thioesterase A [EC:3.1.2.14]	0.65^a^	1.07	1.59^a^	2.37	2.21	1.11
FAB2	acyl-[acyl-carrier-protein] desaturase [EC:1.14.19.2; 1.14.19.11; 1.14.19.26]	0.78	0.95	1.78	1.64	1.71	1.00
GPAT	glycerol-3-phosphate acyltransferase [EC:2.3.1.15]	1.35	1.74^a^	1.17	1.11	3.67^a^	1.29
LPIN	phosphatidate phosphatase LPIN [EC:3.1.3.4]	0.98	0.85	1.50^a^	1.47	1.94^a^	1.12
MGD	1,2-diacylglycerol 3-beta-galactosyltransferase [EC:2.4.1.46]	1.18	0.45^a^	0.49^a^	2.87^a^	0.87	1.32
plcC	phospholipase C [EC:3.1.4.3]	1.09	1.48^a^	1.90^a^	1.76^a^	5.15^a^	0.90

qRT-PCR analysis of cDNA isolated from developing berries at 30, 50 and 70 DAF. The actin gene was used as an internal standard. Values represent the means of three biological triplicates. ^a^indicates a significant difference at 0.05 level

## Discussion

### Differences in key proteins involved in flavonoid synthesis

Flavonoids are secondary metabolites produced from the phenylpropanoid biosynthesis pathway [24]. Since the flavonoid synthesis pathway is a secondary metabolic process with many branches, the concentration of some proteins involved in catalysis is low, which may not be conducive to quantifying all the proteins. Although the flavonoid synthesis pathway is relatively conserved in plants, the differences in the timing or activities of enzymes involved in the synthesis process results in the diversity of flavonoids produced [25]. In this study, we found that most of the enzymes involved in the flavonoid synthesis pathway reached their peak values at 30 DAF, and their concentrations gradually decreased as berry development progressed (Fig. 6). It was speculated that flavonoids accumulated in the pulp of sea buckthorn in the early and middle development stages. Related studies have suggested that PAL, C4H and 4CL play important roles in phenylpropanoid metabolism and flavonoid synthesis in plants [26]. However, phenylpropanoid metabolism is relatively conserved in plants, and PAL, C4H and 4CL do not show significant differences in XE and SJ. CHS is the first key enzyme responsible for the transfer of phenylpropanoid metabolites to flavonoid biosynthesis. Lack of CHS results in no accumulation of flavonoids in *Petunia* *hybrid* [27]. CHS was 1.18 times higher in XE than in SJ at 30 DAF and showed a significant difference at 50 DAF in this study. The difference in CHS concentration between XE and SJ might be the main reason for the difference in total flavonoids between the two cultivars. This result was consistent with the conclusion that the expression of *CHS* was positively correlated with the flavonoid content [28]. Another protein significantly expressed in XE was F3H, which catalyzes the transformation of naringinoids to dihydroflavones and plays an important role in the formation of diverse flavonoids [29]. Previous studies have shown that *F3H* expression in barley promotes the accumulation of flavonoids and anthocyanins, resulting in pigment deposition in tissues [30]. We failed to quantify the changes in the abundance of naringenin 7-O-glucosyltransferase (EC: 2.4.1.185), which competes with F3H, possibly due to its low content. The high expression of *F3H* promoted the synthesis and accumulation of flavonoids and determined the high proportion of rutin in pulp flavonoids, which directly led to the rutin content in the pulp of sea buckthorn being approximately 1000 times higher than that of naringin.

### Differences in key proteins involved in fatty acid and TAG biosynthesis

**Fig. 7:**
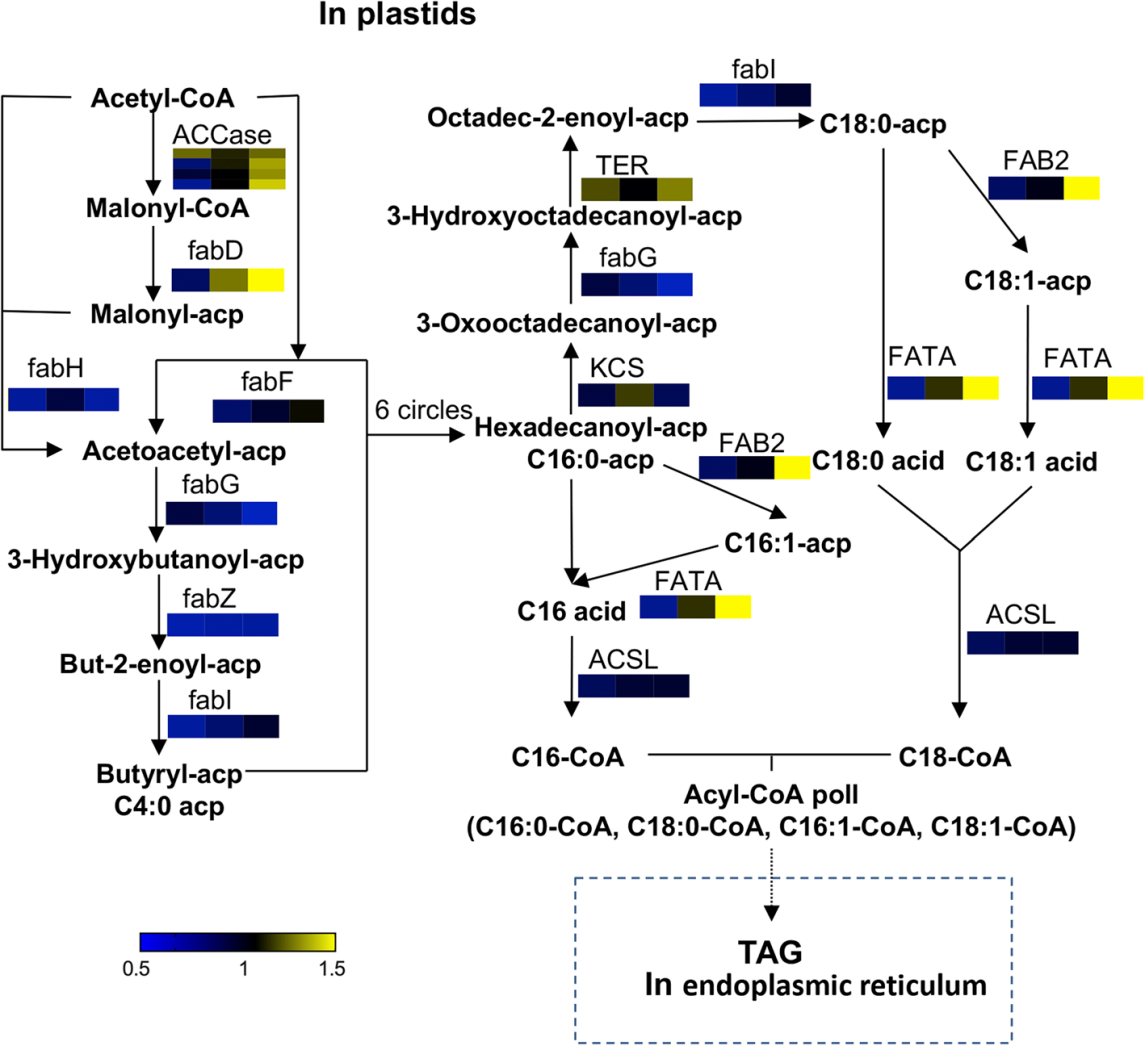
Comparison of key proteins in the fatty acid synthesis pathway between SJ and XE cultivars at different berry developmental stages. The pattern under each enzyme is representative of the ratio of the protein content between SJ and XE cultivars. Left: XE 30 DAF/SJ 30 DAF, middle XE 50 DAF/SJ 50 DAF, right: XE 70 DAF/SJ 70 DAF. This graph was modified from a KEGG map (ko00061) [23]

As fatty acids are the main components of lipids, their synthesis is mainly carried out in plastids [31]. First, ACCase catalyzes acetyl-CoA to form the direct donor of the 2-carbon unit of the fatty acid chain: malonyl-CoA. Then, through the process of condensation, reduction, dehydration and rereduction, the carbon chain is extended by the FAS system (Fig. 7). It is widely believed that ACCase and fabH (KASIII) are the key regulatory enzymes of fatty acid synthesis [32, 33]. The rates of fatty acid synthesis and lipid accumulation in plant seeds are closely related to ACCase. The expression level of *ACCase* in the low-oil mutant *Arabidopsis* was shown to be significantly lower than that in the wild type [34]. The *kas3* gene, formed by amino acid alterations in KASIII, leads to partial loss of the de novo fatty acid synthesis pathway in *Arabidopsis* plastids [35]. In this study, we successfully quantified 4 ACCase subunits and fabH. However, during 50–70 DAF, when the oil contents in XE and SJ pulp were significantly different, the contents of ACCase and fabH did not show significant differences between the two cultivars. These results indicated that the key enzymes ACCase and fabH were not the reasons for the difference in oil content between sea buckthorn cultivars. Meng deduced that fabD (MCT) was one of the key genes leading to high contents of unsaturated fatty acids and oil accumulation in herbaceous peony seed oil [36]. Consistent with their findings, it is worth noting that fabD was significantly upregulated in XE from 50–70 DAF. Both FATA and FAB2 were highly expressed in XE at 50–70 DAF, but their functions influenced the ratio of saturated and unsaturated fatty acids. The protein in the fatty acid synthesis pathway that was most likely responsible for the differences in lipids between SJ and XE was fabD.

**Fig. 8:**
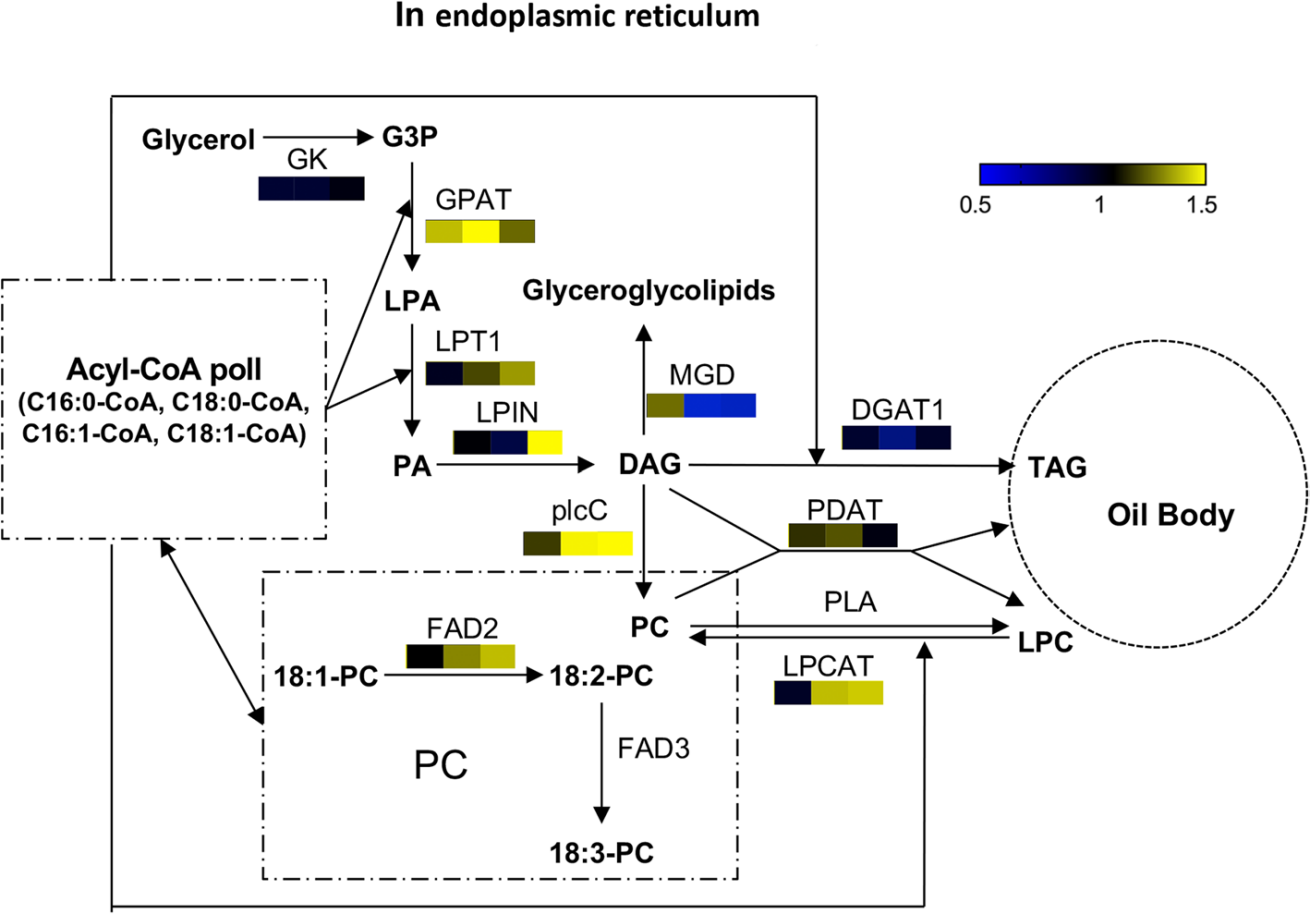
Comparison of key proteins in the triacylglycerol synthesis pathway between SJ and XE cultivars at different berry developmental stages. The pattern under each enzyme is representative of the ratio of the protein content between SJ and XE. Left: XE 30 DAF/SJ 30 DAF, middle XE 50 DAF/SJ 50 DAF, right: XE 70 DAF/SJ 70 DAF. This graph was modified from a KEGG map (ko00561) [23]

TAG synthesis in eukaryotes mainly occurs through the Kennedy pathway: the initio synthesis pathway dependent on acyl CoA, which catalyzes G-3-P and subsequent acylation reactions to produce TAG through related enzymes (Fig. 8). Compared with the fatty acid synthesis pathway in the pulp of sea buckthorn, four enzymes in TAG synthesis showed significant differences between XE and SJ, which mainly appeared in the middle and late development stages. The significantly upregulated proteins were GPAT, LPIN and PLCC, while MGD, which is involved in glycolipid synthesis, was downregulated in the middle and late stages. GPAT synthesizes lysophosphatidic acid by transferring the acyl group of acyl-ACP to the *sn*-1 site of glycerol triphosphate [37], which constitutes the first step in lipid assembly. Misra cloned two *GPAT* genes from *Jatropha* and overexpressed them in *Arabidopsis* *thaliana*, significantly altering the primary metabolism, and the total lipid content increased but the total carbohydrate and soluble protein contents decreased [38]. LPIN and plcC have also been reported to play an important role in TAG synthesis [39–41]. The high content of LPIN in XE pulp at the middle and late stages was conducive to the transformation of PA to DAG, and plcC can cause DAG conversion to TAG from phosphatidylcholine under the condition that DGAT between the two cultivars was not significant (Figs. 7, 8). This phenomenon might be one of the reasons for the difference in oil content between the two cultivars in the middle and late stages of berry development. In the process of lipid synthesis, MGD competes with DGAT and PDAT, converts DAG into glycolipids, and reduces the generation and accumulation of TAG, which could be another reason for the lower oil generation in SJ from 50 to 70 DAF. In contrast to the previously believed decisive role of ACCase, DGAT and PDAT in fatty acid and TAG synthesis pathways [42], in this study, we did not find content differences in these key enzymes between high- and low-oil cultivars, and these key rate-limiting enzymes might not be the reason for the oil content differences between different cultivars of sea buckthorn.

### Oleosins were correlated with oil content

Oleosins are a class of highly hydrophobic, basic low-molecular-weight proteins synthesized from endoplasmic reticulum-bound ribosomes with a molecular weight of approximately 16–26 kDa [43]. These compounds can promote the integrity of oil bodies during cold stress and then improve the cold resistance of seeds so that seeds can still germinate normally after cold stress [44]. This is especially important for sea buckthorn, which can adapt to a wide range of temperatures (-40 ~ 40 °C). In this study, six types of accumulated oleosins were detected in XE and SJ during berry development. Except for oleosins with a weight of 16 kDa, the oleosin content was significantly positively correlated with the oil content at different developmental stages. Subcellular structural localization showed that oleosin with a weight of 16 kDa were enriched in the cytoplasm. Compared with other oleosins enriched in chloroplasts and the plasma membrane, oleosins with a weight of 16 kDa were not the main protein involved in the production of oleosomes. At present, there are still many deficiencies or gaps in the study of oil bodies and oil body proteins; for example, the regulatory mechanism of vegetable oil body proteins in the process of oil body formation is unclear. This knowledge gap can be pursued as a key direction in future research.

## Conclusion

In this study, high-throughput proteomics was used to quantify key enzymes involved in flavonoid synthesis and lipid synthesis pathways in two cultivars at three developmental stages. More than 8600 proteins were identified, among which 6170 proteins were quantified. In the flavonoid synthesis pathway, CHS and F3H were the main enzymes responsible for the difference in flavonoid content between the two cultivars. In the lipid synthesis pathway, LPIN, plcC and MGD were the main enzymes with different contents in the middle to late stages. Higher contents of LPIN and plcC in XE than in SJ could cause DAG to generate TAG from PC, since the difference in DGAT between the two cultivars was not significant. Investigating the causes of flavonoid and oil content differences among different cultivars from the perspective of proteomics could provide a basis for understanding the regulatory mechanism of flavonoids and lipid synthesis in sea buckthorn pulp.

## Methods

Two sea buckthorn cultivars of XE and SJ were chosen among the seedlings of Russia cultivars (*Hippophae* *rhamnoides* ssp. mongolica) planted in Heilongjiang Academy of Agricultural Sciences, Suiling, Heilongjiang, China. The genetic similarity coefficient between two cultivars was 0.756 based on ISSR marker analysis [45]. Characteristics of two cultivars were shown in Fig. S1 and Table S5. The formal identifications of all samples were undertaken by Professor Chengjiang Ruan (Dalian Minzu University). The voucher specimens were deposited at the laboratory of Dalian Minzu University (Dalian, China) under deposition numbers of Xine2020001-3, Suiji2020001-3. The permission was obtained from Heilongjiang Academy of Agricultural Sciences for the collection of sea buckthorn berries. According to our previous observations and studies [1], the berry ripening time of sea buckthorn is about 110–140 days after flowering, during which the berry colour changes from green to yellow-green orange and orange-red. The content of oil and other products gradually stabilized after the color became orange. Berries were collected at three development stages: hard green pulp stage (approx. 30 DAF), green/orange pulp stage (approx. 50 DAF) and orange/red pulp stage (approx. 70 DAF) (Fig. 1). Each sample contains at least 50 berries for subsequent extraction and determination.

### Determination of the oil content in sea buckthorn pulp

The chloroform–methanol method was used to determine the oil content of sea buckthorn pulp at different stages [46]: 5 g freeze-dried pulp powder (M_1_) was transferred to a glass tube, methanol and chloroform (1:2, chromatographically pure, Honeywell) were added for vortexing and ultrasonication for 30 min, then the supernatant was collected and transferred into a new tube, the residue was extracted again with chloroform/methanol solution. The combined supernatant was added to a 1/4 volume of potassium chloride solution (mass concentration: 0.88%), and the bottom layer was collected into a glass sample bottle (M_2_) and then evaporated to a constant weight (M_3_). The oil content was calculated as follows: oil content (%) = (M_3_-M_2_)/M_1_ × 100%. The oil content of each sample was determined for three biological replicates.

### Determination of flavonoids in sea buckthorn pulp

Two grams of sea buckthorn pulp was accurately weighed, combined with 5.0 mL of 75% ethanol, extracted by ultrasonication (210 W) for 10 min, then centrifuged at 4 °C 10,000 r/min for 15 min. The pulp was extracted twice more by the same method, and the supernatants were combined. The supernatant was repeatedly extracted with 10 mL of petroleum ether to remove the pigment. Then, 0.5 mL of the extract was taken, and the volume was fixed with methanol to 5.0 mL. The samples were filtered via 0.22 μm microporous organic for liquid chromatograph mass spectrometer analysis. LC–MS/MS analysis was performed with a DGU-20A liquid chromatograph (Shimadzu Company, Japan) and an electrospray ionization (ESI) 3200 triple quadrupole mass spectrometer (AB SCIEX, USA). The LC conditions were as follows: column, Shimadzu C18 (2.1 mm × 50 mm, 1.9 μm); 0.2 mL/min isocratic elution, 70% methanol with 0.1% formic acid. The sampling time was 10 min, the column temperature was 30 °C, the injection volume was set at 2 μL. MS conditions were as follows: source, electrospray ionization; ionization mode, negative; mass scanning range, 100 ~ 1000 amu; dry gas (N_2_) flow rate, 3 L/min; temperature, 550 °C; curtain gas pressure, 30 psi; and ionization voltage, -4500 V. The scanning mode was multiple response monitoring (MRM) mode. More information about LC–MS/MS parameters and flavonoid standards were shown in Table S3. Three biological replications were performed for each sample.

### Sea buckthorn pulp protein extraction

Fresh sea buckthorn berries after harvesting immediately removed seeds, frozen by liquid nitrogen and grinded into powder. Dissolve pulp powder 2 g in 5 mL centrifuge tube with 3 mL buffer containing 8 mol/L urea, 10 mmol/L dithiothreitol, 35 mg protease inhibitor and 2 mmol/L EDTA, then solution was treated with ultrasound at 4 °C for 5 min: 20 kHz 195 w of intensity, pause 5 s for every 3 s. Centrifuged at 4 °C 20,000 g for 15 min, then supernatant was transferred to a new centrifuge tube, added with 4 mL 15% trichloroacetic acid solution after stand at 20 °C for more than 2 h to precipitate the protein. Centrifuged at 20,000 g 4 °C for 15 min, the protein precipitate was treated by -20 °C acetone for 3 times to remove impurities, added with 8 mol/L urea pH 8.0 with 100 mM tetraethylammonium tetrahydroborate solution to fully dissolve.

### Peptide labeling and LC-MS/MS analysis

Pulp protein was reduced by 10 mmol/L DTT at 40 °C for 1 h then treated with 20 mmol/L IAM (iodoacetamide) for alkylation at 25 °C for 40 min. Protein was digested through two-step method. First, the protein solution was dissolved in triploid 100 mmol/L TEAB solution, then 2 μg trypsin (Roche)/100 μg of protein was added and digested for 11–13 h, then 1 μg trypsin/100 μg of protein was added and digested for about 4 h. The digested peptides were desalted via Strata X C18 SPE column then vacuum dried into powder. Each sample (approximately 100 μg of peptide) was dissolved by adding 0.5 mol/L TEAB, and the peptide labeling procedure was performed according to the Thermo Scientific TMT kit manual. After labelling, peptides were desalted via Strata X C18 SPE column then vacuum dried into powder.

The labeled peptides were reconstructed by solvent A (6% ACN, 0.1% FA) and separated by rapid separation liquid chromatography. The chromatographic column was 50 μm × 150 mm, 2 μm, Thermo Scientific. The gradient elution parameters were: at the flow rate of 400 nL/min, the mobile phase of B (97% acetonitrile, 0.1% formic acid) began from 6 to 22% for 25 min, 22% to 36% for 10 min, and 36% to 85% for 5 min, keep at 85% for 3 min. The peptides were analysed through a Thermo Scientific™ Q Exactive™ and hybrid quadrupole-Orbitrap mass spectrometer. Complete peptides were detected in Orbitrap with a resolution of 70,000. Peptides were extracted by mass spectrometry/mass spectrometry with NCE 30. Ion fragments were detected and selected in Orbitrap at a resolution of 17,400. Dynamic scanning precursor ions of MS were set as: each scanning cycle detected the top 20 precursor ions with the highest response value and the peak value also higher than 10,000. The electrospray voltage was set as 1.5 kV. MS scans range was 350–1800 m/z, automatic gain control: on, fixed first mass: 100 m/z.

### Database search and peptides quantification

Tandem mass spectra data was searched against sea buckthorn (*Hippophae* *rhamnoides* L.) protein database which contain 46,724 sequences. Trypsin was programmed as the digestive enzyme, allowing up to 2 cleavages, 5 modifications and 5 charges for each identified peptide. Other parameters were set as follows: mass error 0.02 Da, difference between fragment and precursor ions 0.01 mg/g, minimum peptide length 7, false discovery rate threshold 7, protein and peptide false discovery rate threshold 1%. For Tandem Mass Tag analysis, the ratios of the reporter ion abundance in MS spectra (range from 126 to 131 m/z, Table S1) from raw data sets were used to analysis content differences between each sample. Protein quantitation was calculated from the median ratio of protein corresponding unique peptides (at least two unique peptides for each protein). The MS proteomic results were uploaded to ProteomeXchange Consortium (http://www.ebi.ac.uk/pride/archive/) via the PRIDE partner repository under dataset identifier PXD009365. Three biological replicates were performed for each sample. Two-sided T-test was performed to determine differences from 0 on the log2 scale and was performed with a unique peptide ratio of each protein. In general, a significance level of 0.05 was used for statistical testing. Two criteria were used to identify significant proteins: (1) fold-change more than 1.5 or lower than 0.67, and (2) p-value less than 0.05.

### Quantitative real-time PCR

Spin Column Plant Total RNA Purification Kit (Sangon, China) was used to extract total RNA from sea buckthorn pulp. The extracted total RNA was reverse transcribed into cDNA using the PrimeScriptTM II 1ST Strand cDNA Synthesis Kit (Takara, Japan). qRT-PCR was performed at LightCycler 480 (Roche, Switzerland) according to the manual of SYBR Premix Ex Taq II (Takara, Japan). The relative expression levels of candidate genes were analyzed by 2^−ΔΔCt^ method, and each sample were repeated for 3 times. qRT-PCR reaction volume was 20 μL, including 2 × SGExcel FastSYBR Mixture 10 μL, PCR primer 2 μL, cDNA 2 μL, RNase free dH_2_O 6 μL. Reaction program: pre-denaturation at 95 °C for 5 s; 60 °C 20 s; 40 cycles; The dissolution curve is 95 °C for 15 s. 60 °C for 1 min; 95 °C 15 s. 60 °C 15 s. qRT-PCR primers were designed using Primer Quest online software (Table S2). Three biological replications were performed for each sample.

## Supplementary Information

**Additional file 1:** **Table S1.** TMT Labeling information.

**Additional file 2:** **Table S2.** Primer sequences used in the PCR experiments.

**Additional file 3:** **Table S3.** LC-MS/MS parameters and quantitative information of flavonoids.

**Additional file 4:** **Table S4.** Information of differentially abundant proteins in flavonoid and lipid synthesis.

**Additional file 5:** **Table S5.** Fruit characteristics of SJ and XE cultivars.

**Additional file 6:** **Figure S1.** Fruit characteristics of XE (A) and SJ (B) cultivars.

**Additional file 7:** **Figure S2.** QC validation of MS data. (A) Mass error distribution of all identified peptides, (B) Peptide length distribution. The distribution of mass error was near zero and most of them are less than 0.1 Da which means the mass accuracy of the MS data fit the requirement. The length of most peptides distributed between 8 and 16, which agree with the property of tryptic peptides, that means sample preparation reach the standard.

## Data Availability

The MS proteomic results were uploaded to ProteomeXchange Consortium (http://www.ebi.ac.uk/pride/archive/) via the PRIDE partner repository under dataset identifier PXD009365 and are publicly available.

## Abbreviations

DAF: Days after flowering
TAG: Triacylglycerol
CHS: Chalcone synthase
F3H: Flavanone 3-hydroxylase
ANS: Anthocyanin synthase
fabD: S-malonyltransferase
FATA: Fatty acyl-ACP thioesterase A
FAB2: Acyl-[acyl-carrier-protein] desaturase
LPIN: Phosphatidate phosphatase LPIN
plcC: Phospholipase C
MGD: 1,2-Diacylglycerol 3-beta-galactosyltransferase
DAG: Diacylglycerol
PC: Phosphatidylcholine
DGAT: Diacylglycerol O-acyltransferase
ACCase: Acetyl-CoA carboxylase carboxyl transferase
PDAT: Phospholipid
PAL: Phenylalanine ammonia-lyase
CYP98A: Coumaroylquinate 3'-monooxygenase
ANR: Anthocyanidin reductase
4CL: 4-Coumarate: CoA ligase
FAS: Fatty acid synthase
PA: Phosphatidic acid
DFR: Dihydroflavonol 4-reductase
LDOX: Anthocyanidin synthase
GPD1: Glycerol-3-phosphate dehydrogenase
G3P: Glycerophosphoric acid
