# Supplementary material for: Identification of the key flavonoid and lipid synthesis proteins in the pulp of two sea buckthorn cultivars at different developmental stages
Source: BMC Plant Biol. 2022 Jun 17;22:299. doi: 10.1186/s12870-022-03688-5 (PMC9205118; doi:10.1186/s12870-022-03688-5)
Supplement: Supplementary file 1 — Additional file 1: Table S1. TMT Labeling information. [file 12870_2022_3688_MOESM1_ESM.docx]

**Table S1.** TMT Labeling information

| **Sample Groups** | **Labeling information** |  |
| --- | --- | --- |
| SJ 30 DAF | 126 |  |
| SJ 50 DAF | 127 |  |
| SJ 70 DAF | 128 |  |
| XE 30 DAF | 129 |  |
| XE 50 DAF | 130 |  |
| XE 70 DAF | 131 |  |
