# Supplementary material for: Identification of the key flavonoid and lipid synthesis proteins in the pulp of two sea buckthorn cultivars at different developmental stages
Source: BMC Plant Biol. 2022 Jun 17;22:299. doi: 10.1186/s12870-022-03688-5 (PMC9205118; doi:10.1186/s12870-022-03688-5)
Supplement: Supplementary file 2 — Additional file 2: Table S2. Primer sequences used in the PCR experiments. [file 12870_2022_3688_MOESM2_ESM.docx]

**Table S2.** Primer sequences used in the PCR experiments.

| **Enzyme Symbol** | **Sequence (5'-3')** | **Product**  **length (bp)** |
| --- | --- | --- |
| CHS | F: AACAAGGTTGCTTTGCTGGT | 180 |
|  | R: CAGCTGCACCATCACCAAAT |  |
| F3H | F: GCTTGTGAAGATTGGGGAGT | 199 |
|  | R: CACGCCAATCTTGAACTGCT |  |
| fabD | F: TGCTGTGTCTGGAGGCTTAA | 166 |
|  | R: TCTGATCTGAGTTGCTGCCA |  |
| FATA | F: CTGATCGTTTGAGGCTTGGG | 166 |
|  | R: TCCAACACCCTGAGCATGAT |  |
| FAB2 | F: GTCAGATGAGAAGCGCCATG | 246 |
|  | R: CACCTGCCAACCAAGAACTC |  |
| GPAT | F: CCAAGAAACTCCTTCACGAAATG | 101 |
|  | R: AGATCGGAGAGTTACGAGGTAT |  |
| LPIN | F: GGGTTCTGTCCAAGCAACTG | 156 |
|  | R: GCCGCAACACACTCACTAAT |  |
| MGD | F: CGGACCAGGTACAATTGCAG | 231 |
|  | R: GCTTCTGGTTGCGCTAGTTT |  |
| plcC | F: TCGTTCCTTTGACCACATGC | 174 |
|  | R: GCTTCAAAAGAGTGACCCGG |  |
| Actin | F: GCAGCAGGTAGTTGAGGATT | 91 |
|  | R: AAGAAGGGCAGAGAACATGAG |  |
