# Supplementary material for: Identification of the key flavonoid and lipid synthesis proteins in the pulp of two sea buckthorn cultivars at different developmental stages
Source: BMC Plant Biol. 2022 Jun 17;22:299. doi: 10.1186/s12870-022-03688-5 (PMC9205118; doi:10.1186/s12870-022-03688-5)
Supplement: Supplementary file 3 — Additional file 3: Table S3. LC-MS/MS parameters and quantitative information of flavonoids. [file 12870_2022_3688_MOESM3_ESM.docx]

**Table S3.** LC-MS/MS parameters and quantitative information of flavonoids.

| **Flavonoids** | **CAS Number** | **Brand Name** | **Q1 Mass (Da)** | **Q3 Mass (Da)** | **DP (Volts)** | **CE (Volts)** | **Retention Time (min)** | **External calibration curve** | **Regression Coefficients （R^2^）** |
| --- | --- | --- | --- | --- | --- | --- | --- | --- | --- |
| Isorhamnetin | 480-19-3 | Sigma | 315 | 300.1 | -35 | -60 | 1.78 | Y=14.5X+99.3 | 0.9996 |
| Quercetin | 117-39-5 | Sigma | 301 | 151 | -29 | -70 | 1.26 | Y=3.22X | 0.9878 |
| Quercetin-3-glucorpyranoside | 482-35-9  482-35-9  482-35-9  482-35-9 | Sigma | 463 | 300 | -28 | -66 | 0.85 | Y=0.0971X+19.6 | 0.9896 |
| Kaempferol | 520-18-3  520-18-3 | Sigma | 285 | 117 | -57 | -77 | 1.66 | Y=29.6X+405 | 0.9994 |
| Epigallocatechin | 970-74-1 | Sigma | 305 | 124.8 | -27 | -47 | 0.68 | Y=95.8X-0.003 | 0.9982 |
| Rutin | 153-18-4 | Shanghai Yuanye | 609.1 | 300 | -50 | -88 | 0.81 | Y=14.4X+9.19 | 0.9984 |
| Gallocatechin gallate | 4233-96-9  4233-96-9 | Sigma | 457 | 169.1 | -24 | -46 | 0.68 | Y=103X+0.003 | 0.9996 |
| Naringenin | 67604-48-2 | Sigma | 271 | 151 | -27 | -50 | 1.30 | Y=72.2X+1410 | 0.9990 |
| Epicatechin gallate | 1257-08-5 | Sigma | 442.3 | 168 | -52 | -57 | 0.70 | Y=0.395X+62.3 | 0.9988 |
| Luteolin | 491-70-3 | Shanghai Yuanye | 284.7 | 133 | -65 | -46 | 1.36 | Y=821X-0.009 | 1 |
| Naringin | 10236-47-2 | Sigma | 579.5 | 150.9 | -80 | -52 | 0.78 | Y=326X+627 | 0.9920 |
| Epicatechin | 490-46-0 | Sigma | 289.1 | 245 | -45 | -50 | 0.73 | Y=0.393X+291 | 0.8762 |
| 2-H Pentahydroxy Flavanone | 480-18-2  480-18-2 | Sigma | 303 | 285 | -45 | -24 | 0.82 | Y=117X+506 | 0.9946 |
| Gallocatechin | 1617-55-6 | Sigma | 305 | 125.2 | -45 | -32 | 0.65 | Y=174X-932 | 0.9998 |
| Dihydromyricetin | 27200-12-0 | Sigma | 319 | 193 | -45 | -50 | 0.71 | Y=7.99X | 0.9942 |
