# Supplementary material for: Identification of the key flavonoid and lipid synthesis proteins in the pulp of two sea buckthorn cultivars at different developmental stages
Source: BMC Plant Biol. 2022 Jun 17;22:299. doi: 10.1186/s12870-022-03688-5 (PMC9205118; doi:10.1186/s12870-022-03688-5)
Supplement: Supplementary file 4 — Additional file 4: Table S4. Information of differentially abundant proteins in flavonoid and lipid synthesis. [file 12870_2022_3688_MOESM4_ESM.docx]

**Table S4.** Information of differentially abundant proteins in flavonoid and lipid synthesis.

|  | **KEGG KO No.** | **Protein description** | **SJ50/30** | **SJ70/50** | **SJ70/30** | **XE50/30** | **XE70/50** | **XE70/30** | **XE30/SJ30** | **XE50/SJ50** | **XE70/SJ70** | **Subcellular Location** |
| --- | --- | --- | --- | --- | --- | --- | --- | --- | --- | --- | --- | --- |
| Flavonoid synthesis pathway | K10775 | PAL; phenylalanine ammonia-lyase [EC:4.3.1.24] | 0.32 | 0.71 | 0.23 | 0.29 | 0.62 | 0.18 | 1.34 | 1.21 | 1.07 | chloroplast |
|  | K00487 | CYP73A; trans-cinnamate 4-monooxygenase [EC:1.14.13.11] | 0.36 | 1.05 | 0.37 | 0.38 | 0.58 | 0.22 | 1.15 | 1.23 | 0.68 | plasma membrane |
|  | K09753 | CCR; cinnamoyl-CoA reductase [EC:1.2.1.44] | 0.51 | 1.18 | 0.60 | 0.46 | 0.81 | 0.37 | 0.87 | 0.78 | 0.53 | chloroplast |
|  | K01904 | 4CL; 4-coumarate--CoA ligase [EC:6.2.1.12] | 1.16 | 0.90 | 1.05 | 1.10 | 0.83 | 0.91 | 0.90 | 0.85 | 0.78 | plasma membrane |
|  | K00660 | CHS; chalcone synthase [EC:2.3.1.74] | 0.63 | 1.01 | 0.64 | 0.43 | 0.78 | 0.34 | 1.87 | 1.29 | 0.99 | cytosol |
|  | K13065 | HCT E2.3.1.133; shikimate O-hydroxycinnamoyltransferase [EC:2.3.1.133] | 0.89 | 0.82 | 0.73 | 0.37 | 0.77 | 0.28 | 1.40 | 0.57 | 0.54 | cytosol |
|  | K09754 | CYP98A; coumaroylquinate(coumaroylshikimate) 3'-monooxygenase [EC:1.14.13.36] | 0.48 | 1.44 | 0.69 | 0.32 | 1.04 | 0.34 | 1.45 | 0.97 | 0.69 | chloroplast |
|  | K00475 | F3H naringenin 3-dioxygenase [EC:1.14.11.9] | 0.36 | 0.55 | 0.20 | 0.40 | 0.31 | 0.12 | 1.55 | 1.71 | 0.95 | cytosol |
|  | K05277 | E1.14.11.19; leucoanthocyanidin dioxygenase [EC:1.14.11.19] | 0.51 | 0.27 | 0.14 | 2.20 | 0.16 | 0.36 | 0.57 | 2.45 | 1.46 | chloroplast |
|  | K08695 | ANR; anthocyanidin reductase [EC:1.3.1.77] | 0.22 | 0.66 | 0.15 | 0.24 | 0.38 | 0.09 | 1.43 | 1.51 | 0.87 | chloroplast |
| Fatty acid synthesis pathway | K11262 | ACACA; acetyl-CoA carboxylase / biotin carboxylase 1 [EC:6.4.1.2 6.3.4.14] | 0.68 | 0.82 | 0.56 | 0.61 | 0.91 | 0.56 | 1.17 | 1.05 | 1.17 | chloroplast |
|  | K01962 | accA; acetyl-CoA carboxylase carboxyl transferase subunit alpha [EC:6.4.1.2] | 1.06 | 0.70 | 0.74 | 1.51 | 0.87 | 1.31 | 0.73 | 1.04 | 1.29 | chloroplast |
|  | K02160 | accB; acetyl-CoA carboxylase biotin carboxyl carrier protein | 1.22 | 0.92 | 1.14 | 1.38 | 1.18 | 1.62 | 0.88 | 0.99 | 1.25 | chloroplast |
|  | K01961 | accC; acetyl-CoA carboxylase, biotin carboxylase subunit [EC:6.4.1.2 6.3.4.14] | 1.10 | 0.81 | 0.89 | 1.70 | 1.13 | 1.92 | 0.64 | 0.99 | 1.38 | chloroplast |
|  | K00645 | fabD; [acyl-carrier-protein] S-malonyltransferase [EC:2.3.1.39] | 1.03 | 0.69 | 0.71 | 1.60 | 0.91 | 1.45 | 0.78 | 1.20 | 1.58 | chloroplast |
|  | K00648 | fabH; 3-oxoacyl-[acyl-carrier-protein] synthase III [EC:2.3.1.180] | 1.41 | 1.56 | 2.19 | 1.93 | 1.09 | 2.13 | 0.62 | 0.86 | 0.60 | chloroplast |
|  | K09458 | fabF; 3-oxoacyl-[acyl-carrier-protein] synthase II [EC:2.3.1.179] | 0.87 | 0.72 | 0.62 | 1.04 | 0.84 | 0.89 | 0.76 | 0.90 | 1.02 | chloroplast |
|  | K15397 | KCS; 3-ketoacyl-CoA synthase [EC:2.3.1.199] | 0.64 | 1.39 | 0.90 | 0.81 | 1.02 | 0.84 | 0.86 | 1.09 | 0.81 | cytosol |
|  | K00059 | fabG; 3-oxoacyl-[acyl-carrier protein] reductase [EC:1.1.1.100] | 1.27 | 1.16 | 1.48 | 1.08 | 0.82 | 0.88 | 0.86 | 0.73 | 0.51 | chloroplast |
|  | K02372 | fabZ; 3-hydroxyacyl-[acyl-carrier-protein] dehydratase [EC:4.2.1.59] | 1.05 | 0.99 | 1.03 | 1.10 | 1.00 | 1.10 | 0.57 | 0.60 | 0.61 | cytosol |
|  | K00208 | fabI; enoyl-[acyl-carrier protein] reductase I [EC:1.3.1.9 1.3.1.10] | 1.21 | 0.97 | 1.18 | 1.50 | 1.17 | 1.75 | 0.61 | 0.75 | 0.90 | chloroplast |
|  | K10258 | TER; very-long-chain enoyl-CoA reductase [EC:1.3.1.93] | 1.29 | 0.81 | 1.04 | 1.13 | 0.99 | 1.12 | 1.12 | 0.98 | 1.22 |  |
|  | K10782 | FATA; fatty acyl-ACP thioesterase A [EC:3.1.2.14] | 1.05 | 0.77 | 0.82 | 1.63 | 1.10 | 1.67 | 0.65 | 1.07 | 1.59 | chloroplast |
|  | K03921 | FAB2; acyl-[acyl-carrier-protein] desaturase [EC:1.14.19.2 1.14.19.11 1.14.19.26] | 1.23 | 0.68 | 0.83 | 1.50 | 1.26 | 1.89 | 0.78 | 0.95 | 1.78 | chloroplast |
|  | K01897 | ACSL; long-chain acyl-CoA synthetase [EC:6.2.1.3] | 1.69 | 1.33 | 2.24 | 1.86 | 1.34 | 2.51 | 0.80 | 0.89 | 0.90 | chloroplast |
| Triacylglycerol synthesis pathway | K00864 | GK glpK; glycerol kinase [EC:2.7.1.30] | 0.77 | 0.73 | 0.56 | 0.77 | 0.78 | 0.61 | 0.89 | 0.89 | 0.96 | cytosol |
|  | K13508 | GPAT; glycerol-3-phosphate acyltransferase [EC:2.3.1.15] | 0.34 | 1.03 | 0.35 | 0.43 | 0.69 | 0.30 | 1.35 | 1.74 | 1.17 | plasma membrane |
|  | K13519 | LPT1; lysophospholipid acyltransferase [EC:2.3.1.51 2.3.1.23 2.3.1.-] | 0.90 | 1.01 | 0.90 | 1.07 | 1.16 | 1.24 | 0.93 | 1.11 | 1.27 | plasma membrane |
|  | K15728 | LPIN; phosphatidate phosphatase LPIN [EC:3.1.3.4] | 1.37 | 0.60 | 0.83 | 1.22 | 0.94 | 1.26 | 0.98 | 0.85 | 1.50 | nuclear |
|  | K10256 | FAD2; omega-6 fatty acid desaturase / acyl-lipid omega-6 desaturase (Delta-12 desaturase) [EC:1.14.19.6 1.14.19.22] | 5.13 | 0.28 | 1.43 | 5.95 | 0.28 | 1.93 | 1.00 | 1.23 | 1.35 | plasma membrane |
|  | K11155 | DGAT1; diacylglycerol O-acyltransferase 1 [EC:2.3.1.20 2.3.1.75 2.3.1.76] | 1.25 | 1.18 | 1.47 | 0.96 | 1.55 | 1.49 | 0.90 | 0.69 | 0.91 | plasma membrane |
|  | K00679 | PDAT E2.3.1.158; phospholipid:diacylglycerol acyltransferase [EC:2.3.1.158] | 0.82 | 1.07 | 0.88 | 0.87 | 0.91 | 0.79 | 1.07 | 1.13 | 0.96 | nuclear |
|  | K03715 | MGD E2.4.1.46; 1,2-diacylglycerol 3-beta-galactosyltransferase [EC:2.4.1.46] | 1.94 | 1.18 | 2.28 | 0.73 | 1.28 | 0.93 | 1.18 | 0.45 | 0.49 | cytoskeleton |
|  | K01114 | plcC; phospholipase C [EC:3.1.4.3] | 0.76 | 0.72 | 0.55 | 1.03 | 0.94 | 0.96 | 1.09 | 1.48 | 1.90 |  |
|  | K01152 | LPCAT1_2; lysophosphatidylcholine acyltransferase / lyso-PAF acetyltransferase [EC:2.3.1.23 2.3.1.67] | 1.13 | 1.20 | 1.35 | 1.67 | 1.21 | 2.01 | 0.92 | 1.35 | 1.38 |  |
| Oil body |  | 92 580 minus strand oleosin 2 [Jatropha curcas] | 5.33 | 1.06 | 5.67 | 6.33 | 2.19 | 14.18 | 0.74 | 0.90 | 1.87 | plasma membrane |
|  |  | 44 484 minus strand PREDICTED: oleosin 5-like [Solanum lycopersicum] | 7.94 | 0.76 | 6.01 | 5.60 | 1.47 | 8.30 | 1.19 | 0.83 | 1.61 | chloroplast |
|  |  | 61 564 minus strand PREDICTED: oleosin 16 kDa-like [Fragaria vesca subsp. vesca] | 2.90 | 0.84 | 2.43 | 2.10 | 1.35 | 2.83 | 1.65 | 1.19 | 1.92 | chloroplast |
|  |  | 71 499 PREDICTED: oleosin 16 kDa [Vitis vinifera] | 4.45 | 0.97 | 4.33 | 7.33 | 0.69 | 5.14 | 0.75 | 1.30 | 0.92 | cytosol |
|  |  | 1 273 minus strand PREDICTED: oleosin 18.2 kDa-like [Fragaria vesca subsp. vesca] | 3.90 | 0.91 | 3.66 | 4.14 | 1.62 | 6.58 | 0.91 | 0.93 | 1.65 | chloroplast |
|  |  | 108 515 minus strand oleosin L-isoform [Ficus pumila var. awkeotsang] | 3.13 | 0.75 | 2.55 | 3.54 | 1.94 | 6.85 | 0.83 | 0.83 | 2.21 | chloroplast |
