# Supplementary material for: Identification of the key flavonoid and lipid synthesis proteins in the pulp of two sea buckthorn cultivars at different developmental stages
Source: BMC Plant Biol. 2022 Jun 17;22:299. doi: 10.1186/s12870-022-03688-5 (PMC9205118; doi:10.1186/s12870-022-03688-5)
Supplement: Supplementary file 5 — Additional file 5: Table S5. Fruit characteristics of SJ and XE cultivars. [file 12870_2022_3688_MOESM5_ESM.docx]

| Cultivar | Fruit moisture content（%） | Fruit size（cm）  Vertical diameter Transverse diameter | | Fruit color | Yield per plant  （kg） | Fruit juice BRIX | Malic acid（%） | Sugar-acid ratio |
| --- | --- | --- | --- | --- | --- | --- | --- | --- |
| Suiji 1 | 87.27 | 1.30 | 0.78 | Light orange | 3.50 | 7.05 | 2.26 | 3.13 |
| Xin‘e 3 | 85.19 | 1.27 | 0.99 | Vermilion | 5.40 | 6.58 | 1.76 | 3.74 |

**Table S5**. Fruit characteristics of SJ and XE cultivars.
