# Supplementary figures and images for: Identification of the key flavonoid and lipid synthesis proteins in the pulp of two sea buckthorn cultivars at different developmental stages
Source: BMC Plant Biol. 2022 Jun 17;22:299. doi: 10.1186/s12870-022-03688-5 (PMC9205118; doi:10.1186/s12870-022-03688-5)

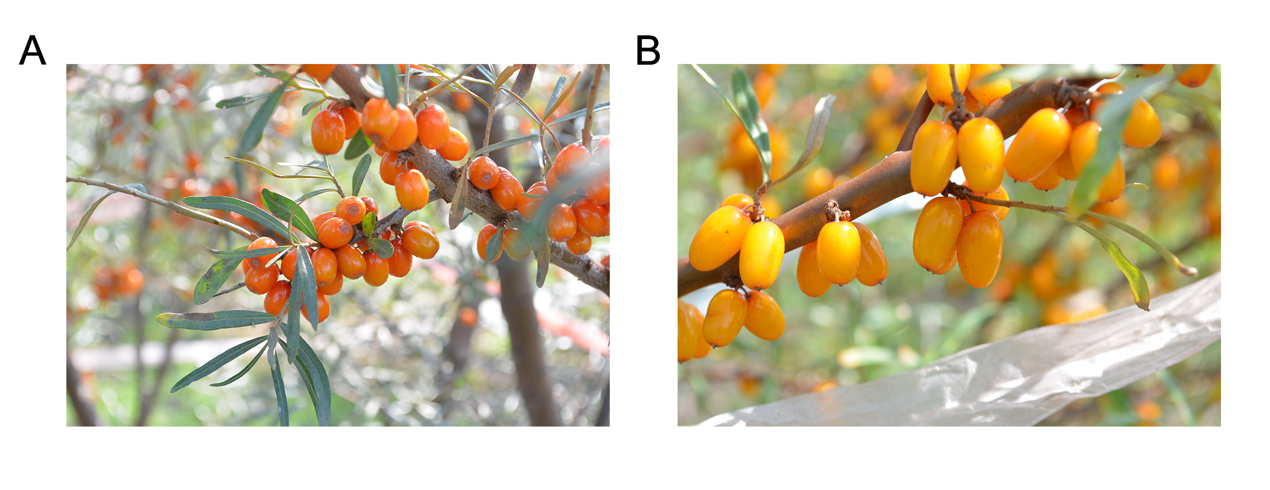


**Fig. S****1** Fruit characteristics of XE (A) and SJ (B) cultivars.

Supplement: Supplementary file 6 — Additional file 6: Figure S1. Fruit characteristics of XE (A) and SJ (B) cultivars. [file 12870_2022_3688_MOESM6_ESM.docx]
