# Supplementary material for: Identification of the key flavonoid and lipid synthesis proteins in the pulp of two sea buckthorn cultivars at different developmental stages
Source: BMC Plant Biol. 2022 Jun 17;22:299. doi: 10.1186/s12870-022-03688-5 (PMC9205118; doi:10.1186/s12870-022-03688-5)
Supplement: Supplementary file 7 — Additional file 7: Figure S2. QC validation of MS data. (A) Mass error distribution of all identified peptides, (B) Peptide length distribution. The distribution of mass error was near zero and most of them are less than 0.1 Da which means the mass accuracy of the MS data fit the requirement. The length of most peptides distributed between 8 and 16, which agree with the property of tryptic peptides, that means sample preparation reach the standard. [file 12870_2022_3688_MOESM7_ESM.docx]

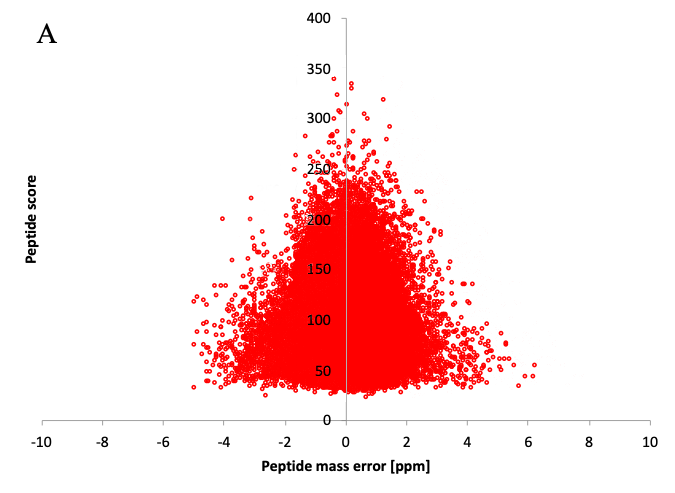

**Fig. S****2**  QC validation of MS data. (A) Mass error distribution of all identified peptides, (B) Peptide length distribution. The distribution of mass error was near zero and most of them are less than 0.1 Da which means the mass accuracy of the MS data fit the requirement. The length of most peptides distributed between 8 and 16, which agree with the property of tryptic peptides, that means sample preparation reach the standard.
